# Supplementary material for: A Humanized Yeast Phenomic Model of Deoxycytidine Kinase to Predict Genetic Buffering of Nucleoside Analog Cytotoxicity
Source: Genes (Basel). 2019 Sep 30;10(10):770. doi: 10.3390/genes10100770 (PMC6826991; doi:10.3390/genes10100770)
Supplement: Supplementary file 1 [file genes-10-00770-s001.zip › Additional_Files/Additional_File7_GOTermHeatmaps.zip!/Additional_File7_GOTermHeatmaps/transcription_elongation_factor_complex.pdf]

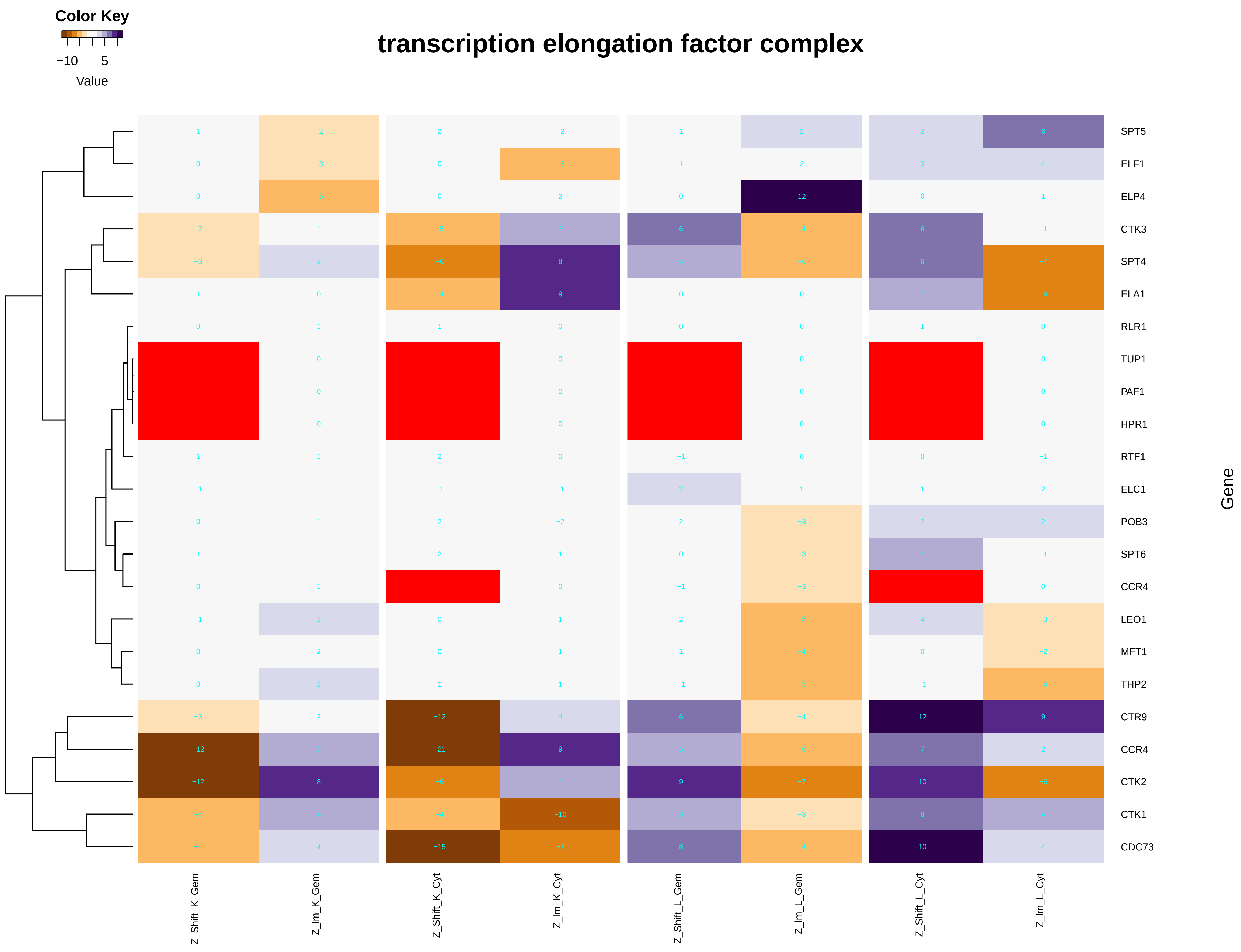
Color Key
−10 5
Value
transcription elongation factor complex
SPT5
ELF1
ELP4
CTK3
SPT4
ELA1
RLR1
TUP1
PAF1
HPR1
RTF1
ELC1
POB3
SPT6
CCR4
LEO1
MFT1
THP2
CTR9
CCR4
CTK2
CTK1
CDC73
Gene
Z_Shift_K_Gem
Z_Im_K_Gem
Z_Shift_K_Cyt
Z_Im_K_Cyt
Z_Shift_L_Gem
Z_Im_L_Gem
Z_Shift_L_Cyt
Z_Im_L_Cyt

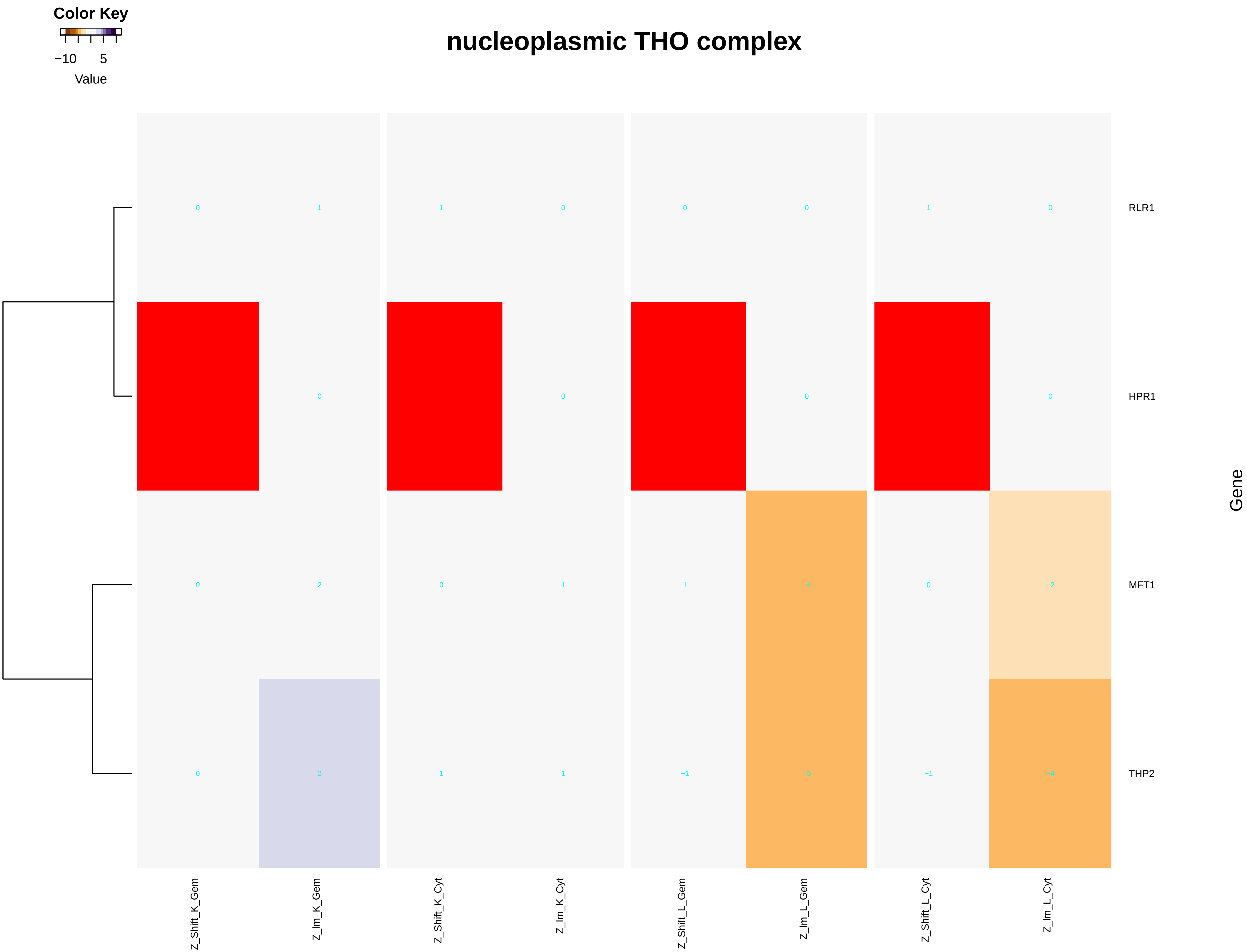

Color Key
−10
5
Value
nucleoplasmic THO complex
RLR1
HPR1
MFT1
THP2
Gene
Z_Shift_K_Gem
Z_Im_K_Gem
Z_Shift_K_Cyt
Z_Im_K_Cyt
Z_Shift_L_Gem
Z_Im_L_Gem
Z_Shift_L_Cyt
Z_Im_L_Cyt

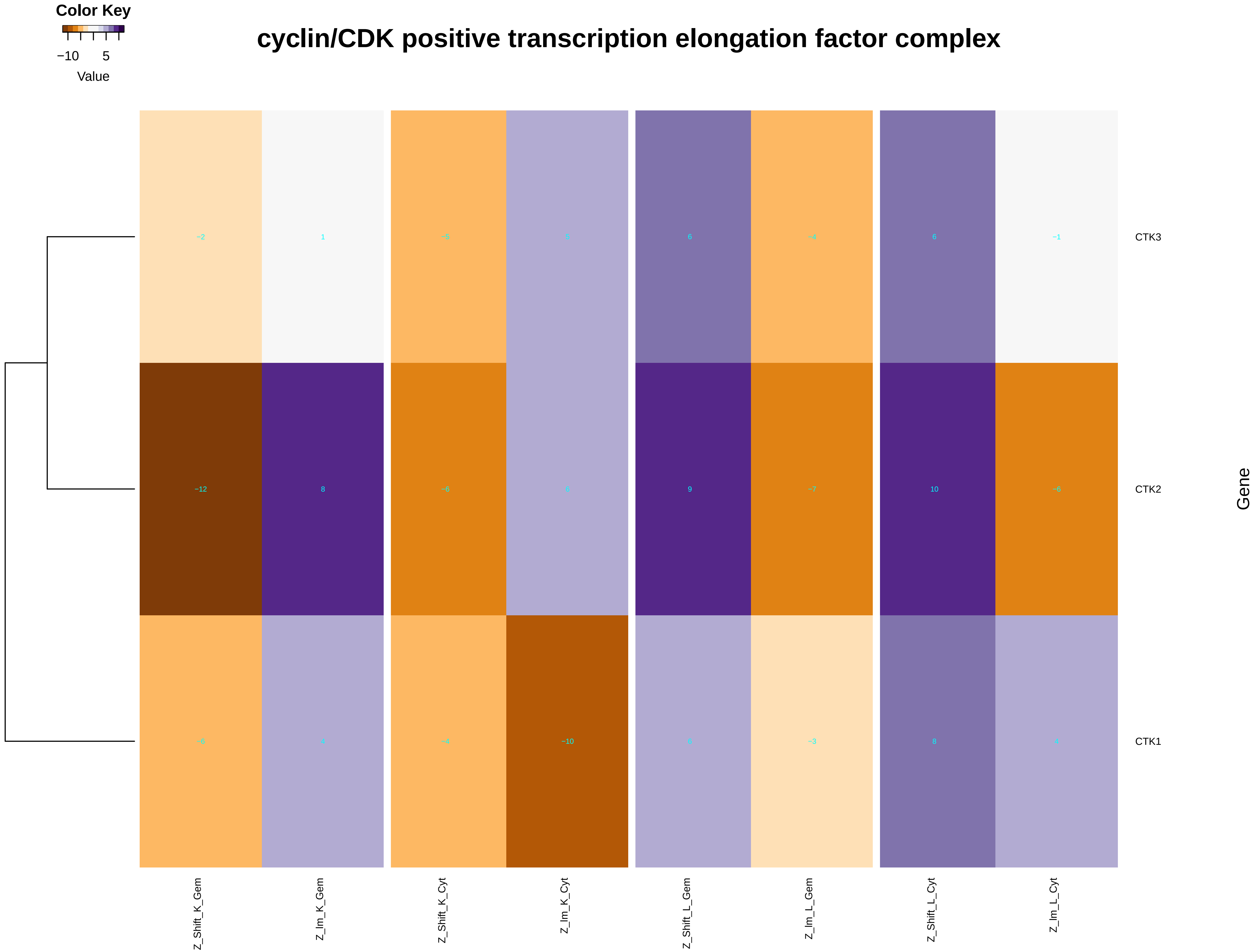

Color Key
−10
5
Value
cyclin/CDK positive transcription elongation factor complex
−2
1
−5
5
6
−4
6
−1
CTK3
−12
8
−6
6
9
−7
10
−6
CTK2
−6
4
−4
−10
6
−3
8
4
CTK1
Gene
Z_Shift_K_Gem
Z_Im_K_Gem
Z_Shift_K_Cyt
Z_Im_K_Cyt
Z_Shift_L_Gem
Z_Im_L_Gem
Z_Shift_L_Cyt
Z_Im_L_Cyt

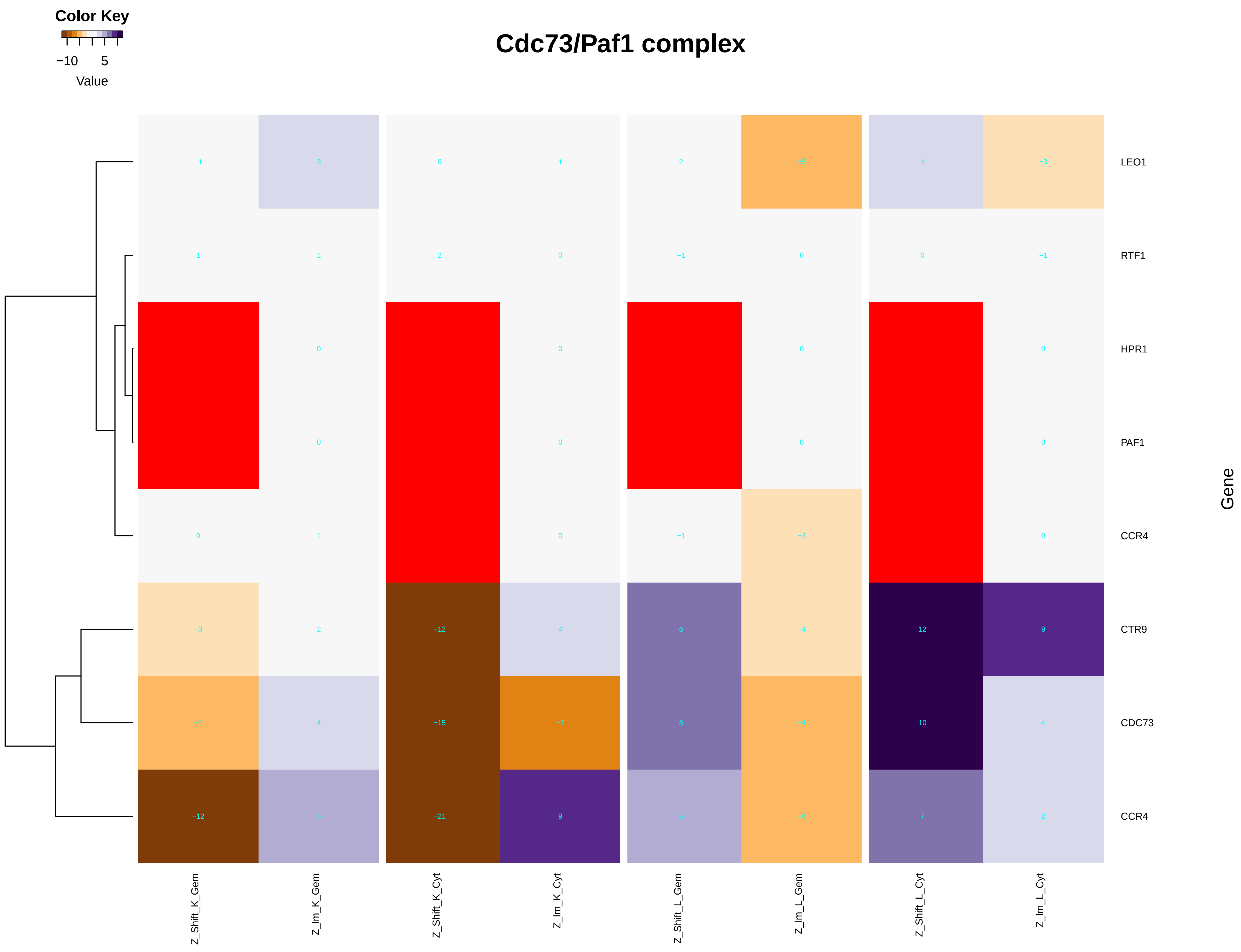

Color Key
−10
5
Value
Cdc73/Paf1 complex
LEO1
RTF1
HPR1
PAF1
CCR4
CTR9
CDC73
CCR4
Gene
Z_Shift_K_Gem
Z_Im_K_Gem
Z_Shift_K_Cyt
Z_Im_K_Cyt
Z_Shift_L_Gem
Z_Im_L_Gem
Z_Shift_L_Cyt
Z_Im_L_Cyt

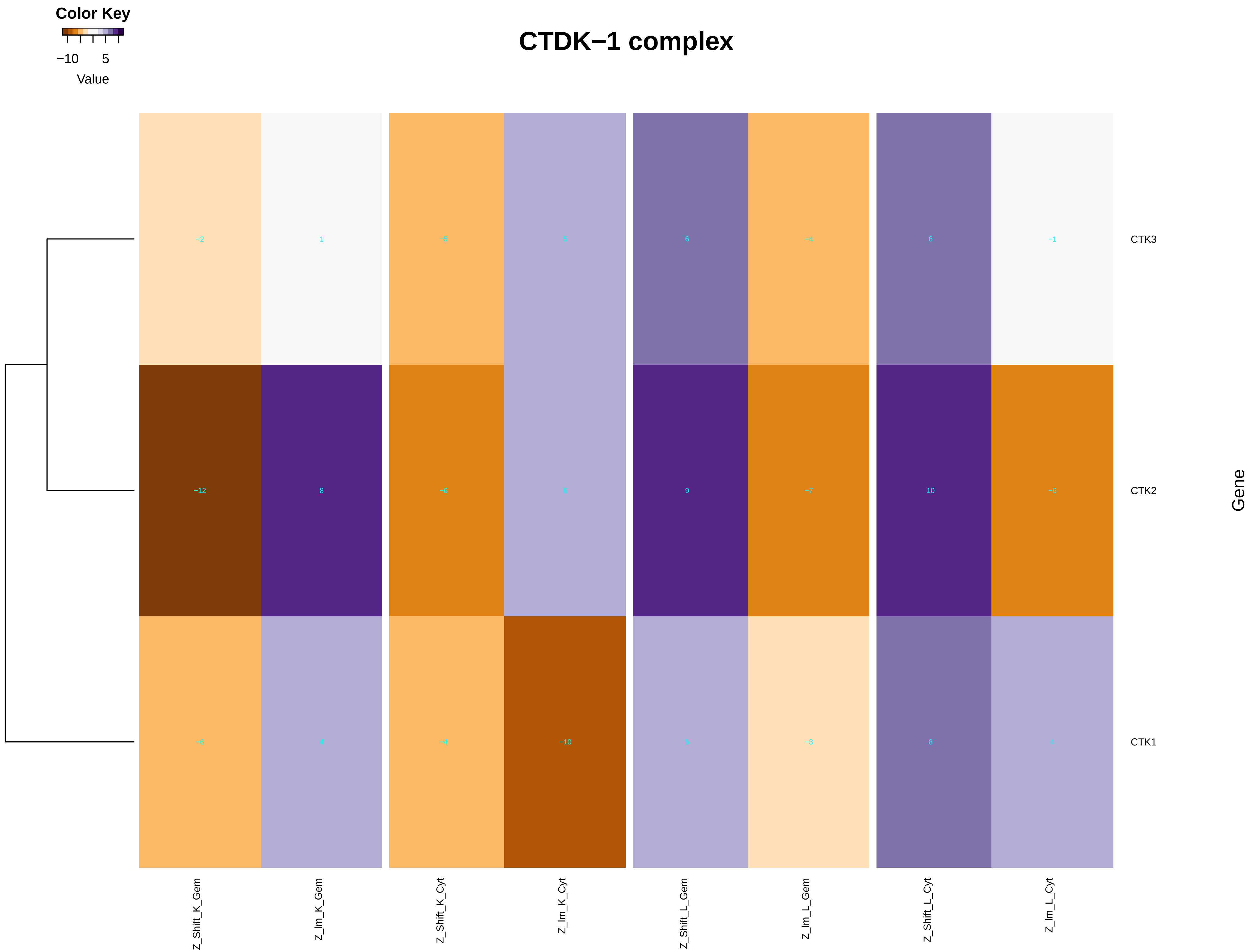

Color Key
−10
5
Value
CTDK−1 complex
−2
1
−5
5
6
−4
6
−1
CTK3
−12
8
−6
6
9
−7
10
−6
CTK2
−6
4
−4
−10
6
−3
8
4
CTK1
Gene
Z_Shift_K_Gem
Z_Im_K_Gem
Z_Shift_K_Cyt
Z_Im_K_Cyt
Z_Shift_L_Gem
Z_Im_L_Gem
Z_Shift_L_Cyt
Z_Im_L_Cyt
